# Wants to Study Medicine

**Published:** 1894-09

**Authors:** 


### WANTS TO STUDY MEDICINE.

The following letter was placed in our hands a few days ago. The applicant is too late. The time has now passed, we think, when doctors will be made of such material:

---

AUG THE 14th, 1894.

Dr. ———————— dane Sir please send us a Catlog of the ———————— ———— College We hav bin Contemplating entring College this fall Some Wheare and if you Will give us as short a time as We Can get enyWhear and as Cheep pobly we Will enter yoars College but if We Can dow Beter Some Whea els We Will do so We air pooar in this worlds goods We think yoar tearms air tow Meny for us as we air a geting up in years We Caint spend so mutch time.

adress ————————

